# *Caenocentron* Schmid, 1982 (Trichoptera: Xiphocentronidae), a New Species from Mexico, Genetic Diversity, and an Updated Identification Key

**DOI:** 10.1007/s13744-026-01409-3

**Published:** 2026-07-28

**Authors:** María Razo-González, Albane Vilarino, Yokomi Nisei Lozano-Sardaneta, Silvio Shigueo Nihei, Atilano Contreras-Ramos

**Affiliations:** 1 Colección Nacional de Insectos, Depto de Zoología, Instituto de Biología, Univ Nacional Autónoma de México, Ciudad de Mexico, Mexico; 2 Depto de Zoologia, Instituto de Biociências, Univ de São Paulo, Cidade Universitária, São Paulo, Brazil

**Keywords:** Aquatic insects, Caddisflies, Xiphocentroninae, Taxonomy, COI barcode, Morphology

## Abstract

*Caenocentron* Schmid, 1982 is a small net-tube caddisfly genus distributed in the southern Nearctic and Neotropical regions. Two species were previously recorded in Mexico: *Caenocentron trilineatum* (Mosely 1934) and *Caenocentron ideolus* Schmid, 1982. In this study, *Caenocentron chinantecum* Razo-González and Vilarino, sp. nov. is described from Oaxaca, Mexico, which is similar to *C. carlosdelarosai* Vilarino, Dias and Bispo, 2022 from Costa Rica, with main differences observed in the shape of inferior appendage, position of its spine, and the paraproct lateral spine-like setae and asymmetrical dorsal processes. COI barcode of the new species and congeners was compared and the intra and interspecific distances were assessed. Minimum interspecific differences in the genus were around 5%, which may serve as a threshold for species delimitation. ASAP, PTP, TCS, and ABGD species delimitation models were used, but only the last one was congruent with morphological species criteria. Maximum likelihood analysis placed the new species closely related to *C. carlosdelarosai.* The haplotype structure of the species showed 26 haplotypes. *Caenocentron carlosdelarosai* showed eight haplotypes, clustering in two main groups with a high genetic distance (4.6%) but without morphological differences. An updated identification key for *Caenocentron* species is provided.

## Introduction

Distributed across the World tropical zones, Xiphocentronidae Ross, 1949 are a family of net-tube caddisflies comprising around 200 species (Vilarino and Bispo 2020). Adults have typically a small size (2–5 mm forewing length, but with *X. aureum* Flint, 1967 reaching 8.5 mm), are often active during the day, and rarely collected in larger numbers (Flint 1968; Schmid 1982). Their larvae construct silken tunnels in hygropetric environments and feed on surface biofilm (Sturm 1960; Muñoz-Quesada and Holzenthal 1997; Pes et al. 2013). The family is organized into three subfamilies: Xiphocentroninae Schmid, 1982 (including seven genera), Proxiphocentroninae Schmid, 1982 (with one Oriental genus) and Palerasnitsyninae Wichard 2023 (with one Cretaceous fossil genus, †*Palerasnitsynus*). Xiphocentronidae in the New World are represented by three genera: *Caenocentron* Schmid, 1982 and *Machairocentron* Schmid, 1982, each including 10 species; and *Xiphocentron* Brauer, 1870 with 68 species, occurring from the southern United States to northern Argentina.

**Fig. 1:**
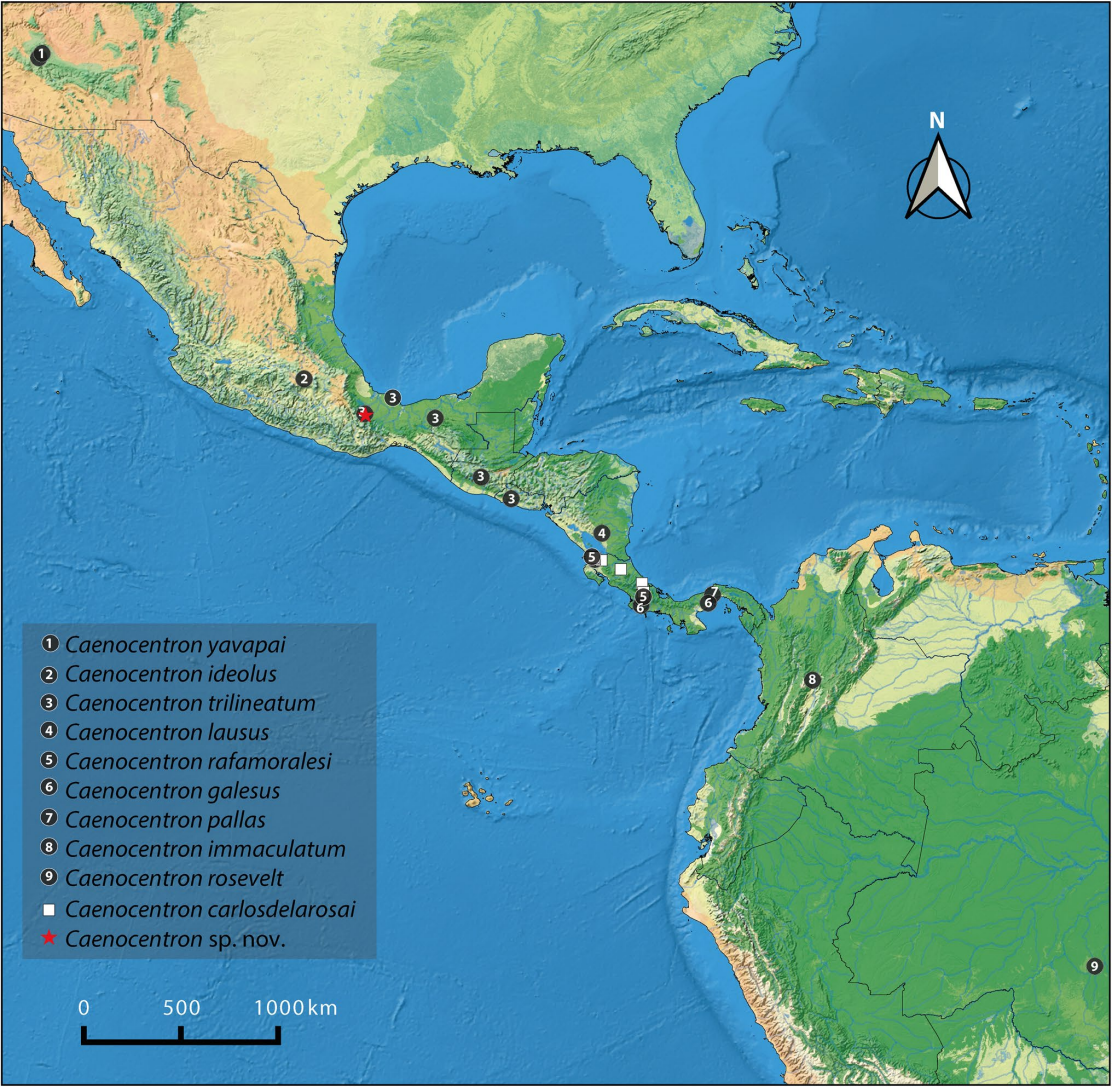
*Caenocentron* Schmid, 1982 species distribution

*Caenocentron* was previously a subgenus of *Cnodocentron* Schmid, 1982, which included species from the Americas and Southeast Asia (Schmid 1982), both with male genitalia bearing a projection on the coxopodite. However, *Caenocentron* was phylogenetically more closely related to other Neotropical genera than to the Oriental *Cnodocentron*, therefore, it was elevated to genus level (Vilarino et al. 2022). Currently, 10 species of *Caenocentron* are found from the southern United States (Nearctic region), throughout Mesoamerica, northern Colombia (South America), and the most recently described species from the Brazilian Amazon (Desiderio et al. 2025) (Fig. 1). The genus was thought to have its early radiation in Mesoamerica, subsequently reaching South America around 8 to 10 mya (Vilarino et al. 2022); however, the species from the Amazon suggests a more complex scenario with an early presence in South America (Desiderio et al. 2025). *Caenocentron* is characterized by the synapomorphies: preanal appendage slender, basal plate apodeme directed ventrad, and coxopodite caudal margin produced posterad (Desiderio et al. 2025).

Two *Caenocentron* species were previously recorded in Mexico, *C. trilineatum* (Mosely 1934) with several records in southern Mexico (states of Veracruz, Tabasco, and Oaxaca), also with records in Guatemala and El Salvador (Holzenthal and Calor 2017), and *C. ideolus* Schmid, 1982 with a single record from the holotype locality, which was collected by Dr. Alfonso Dampf (1884–1948) probably in the 1940 s (collection date was not provided) in Lomas de Chapultepec, in the metropolitan area of Mexico City (Schmid 1982).

In this study, the description and illustration of a new species of *Caenocentron* from Oaxaca, Mexico, are provided. Additionally, cytochrome c oxidase I (COI) barcode fragments of the new species and other congeners were used to confirm their taxonomic placement, as well as their genetic distance and haplotype structure were assessed in order to determine the degree of differentiation of the species.

## Materials and Methods

### Specimen Collection, Identification, and Description

**Fig. 2:**
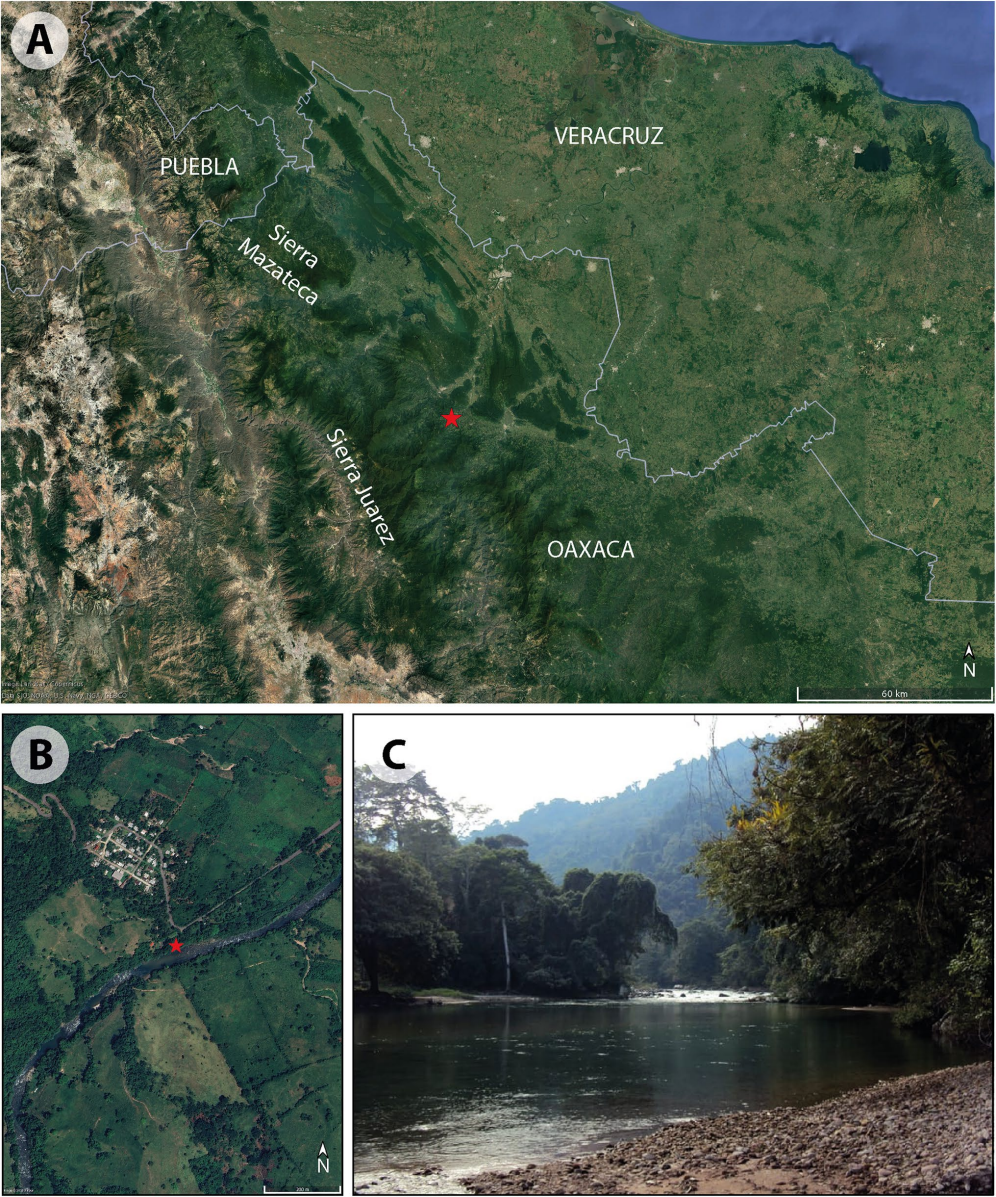
*Caenocentron chinantecum* Razo-González and Vilarino, sp. nov. collection site: **A** satellite image from Google Earth® showing Sierra de Juárez and type locality; **B** new species collection site at San Martín Soyolapam; **C** image of the river bank at the collection site

Field work was in Sierra de Juárez, a mountain range in north-central Oaxaca, Mexico, in one of the tributaries of the Valle Nacional river (Fig. 2). Collecting was made through light-trap between 7:30 and 8:00 pm. The specimen collected was directly preserved in 96% ethanol. To examine the male genital structures, the abdomen of the specimen was removed and subjected to clearing using hot 10% KOH, following the detailed procedures outlined by Blahnik and Holzenthal (2004). Subsequent to the clearing process, the abdomen was mounted on a temporary excavated slide with a drop of a mixture of 60% glycerin and 40% alcohol gel hand sanitizer (to achieve optimum viscosity and prevent the abdomen from floating when in the anatomic position) and examined under a compound microscope. The abdomen was subsequently stored in a microvial with ethanol, along with the remains of the respective specimen placed in a vial with 96% ethanol.

Images of the specimen were captured using a digital camera attached to a stereo microscope. Multiple images of each structure were obtained at different focal distances and then stacked and combined into a single image using the Helicon Focus® stacking software. Male genitalia were photographed using a camera attached to a compound microscope, and the stacked images served as templates for creating vector graphic illustrations in Adobe Illustrator®, aided by a graphic tablet and pen.

Distribution maps were generated using QGIS software (version 2.8.2), incorporating the terrestrial ecoregion shapefile from One Earth (2023), based on the classification of Olson et al. (2001). Satellite imagery was obtained from Google Earth®. Distribution data were compiled from literature sources and examined specimens from the insect collection of Universidad Nacional Autónoma de México, distribution data of *Caenocentron trilineatum* not previously informed is listed in the taxonomic section.

Male genitalia terminology is based on Nielsen (1957) and Schmid (1982), as interpreted for *Caenocentron* by Vilarino et al. (2022). Wing venation terminology follows the Comstock-Needham system, as applied to Trichoptera by Mosely and Kimmins (1953). The holotype is deposited in the Colección Nacional de Insectos of the Instituto de Biología (CNIN) at Universidad Nacional Autónoma de México (UNAM), Ciudad de México, Mexico.

### DNA Extraction, Amplification, Sequencing, and Alignments

Genomic DNA was extracted through nondestructive methods using the whole specimen body with detached abdomen; the tissue was incubated overnight for lysis in a solution of Chelex-100 at 10% and Proteinase K following the protocol of Lozano-Sardaneta et al. (2021). Because DNA amplification failed with the standard Folmer et al. (1994) barcode primers, the partial sequence of mitochondrial COI (DNA barcode) was amplified in two shorter fragments with the combination of the primers forward: C1-J-1718 (5-GAG GAT TTG GAA ATT GAT TAG TTC C-3) (Simon et al. 1994) and reverse: HCO-2198 (5-TAA ACT TCA GGG TGA CCA AAA AAT CA-3) (Folmer et al. 1994); and the forward: cocktail C_LepFo1F = LepF1 (5- ATT CAA CCA ATC ATA AAG ATA TTG G-3) (Hebert et al. 2003) + LCO1490 (5-GGT CAA CAA ATC ATA AAG ATA TTG G-3) (Folmer et al. 1994), and reverse: MLepR3 (5-GCT AAR TGW ARD GAA AAA ATW GC-3) (Hernández-Triana et al. 2014). Protocols for PCR conditions consisted of 3 min at 94 °C, followed by 5 cycles of 30 s at 94 °C, 30 s at 45 °C, and 1 min at 72 °C, and 35 cycles of 30 s at 94 °C, 30 s at 48 °C, and 1 min at 72 °C with a final step of 7 min at 72°C. PCR mixture was prepared at 20 µL with 10 µL GoTaq Green Master Mix (Promega, Madison, USA), 1 µL of each primer (100 ng), 1 µL of Bovine serum albumin (BSA), 2 µL DNA (~20 ng/µL), and 5 µL nuclease-free water. Electrophoresis was performed in 1.5% agarose gel stained with 0.2 µL Midori Green Advance DNA stain (Nippon Genetics Europe). PCR products were visualized under UV light. Purification and sequencing of PCR products took place at Laboratorio de Secuenciación Genómica de la Biodiversidad y de la Salud, Instituto de Biología, UNAM. Chromas version 2.6.6 (http://technelysium.com.au/) was used for the visualization of electropherograms. The sequences generated as part of this study were deposited in GenBank under accessions PX837064 (*Caenocentron chinantecum* sp. nov.), PX837065 (*Caenocentron trilineatum*).

### Phylogenetic Analyses and K2P Divergences

The mitochondrial COI sequence fragments obtained for the new species, *C. trilineatum*, and the data of the *Caenocentron* species previously available at the BOLD website (Barcode of Life Database) were used to infer their genetic relationship. The specimens were considered in the same species when they were clustered in the same clade, following the Phylogenetic Species Concept (Eldredge and Cracraft 1980), and when some consistent diagnosable morphological evidence was available. The sequences were aligned using the ClustalW algorithm in MEGA X (Kumar et al. 2018). The substitution model was selected considering the Akaike Information Criterion (AIC) (Akaike 1974). The GTR+I+G evolution model was selected. A maximum likelihood phylogenetic analysis was performed in MEGA X with 1000 non-parametric bootstraps replicates used to test support for tree nodes (Felsenstein 1985). The tree was rooted with *C. rafamoralesi* Vilarino et al. 2022 according to previous phylogenies, which suggest it is an ancestral lineage (Vilarino et al. 2022). Intra- and interspecific genetic distances were computed using the Kimura 2-parameter (K2P) model (Kimura 1980) in Mega X.

The following molecular methods for species delimitation were used: Automatic Barcode Gap Discovery (ABGD) (Puillandre et al. 2012); Assemble Species by Automatic Partitioning (ASAP) (Puillandre et al. 2021); Poisson Tree Processes (PTP) (Zhang et al. 2013); and TCS haplotype network (https://bioresearch.byu.edu/tcs/) (Clement et al. 2002). The analyses ABGD and ASAP were performed in the online server SPART (https://spartexplorer.mnhn.fr/) (Miralles et al. 2022). The analyses were run using the K2P model with default settings; the ABGD a priori minimum distance value was set to 3%, since one-third of the Trichoptera species show COI intraspecific variation greater than 3% (Zhang and Bu 2022), other settings were used in default. The PTP model was running on the web server https://mptp.h-its.org/, using single-rate methods. For TCS, the alignment was used as an input file using the default parameters.

The number of haplotypes and average number of nucleotide differences were calculated using DnaSP v5.10 (Librado and Rozas 2009). Fixation index (FST) was considered to differentiate the genetic structure of the species using DnaSP. It contrasts the genetic variation within populations against the total genetic variance among all populations, yielding a number from 0 (indicating little distinction) to 1 (indicating perfect differentiation) (Wright 1984). PopART (http://popart.otago.ac.nz/) was used to construct a haplotype network graph, through TCS Networks to estimate gene genealogies (Clement et al. 2002).

## Results

### New Species Placement and *Caenocentron *Genetic Diversity

Intraspecific genetic distance is indicated in Table 1, with the maximum distance ranged between 3 and 5%, and a mean of 1–2% in the species with better sample size. Genetic distance between species is indicated in Table 2, where the minimum distance between species is around 5% and the mean interspecific distance ranging from 6 to 11%. The degree of the species differentiation according to the FST is shown in Table 3.

**Table 1:** Intraspecific genetic distance, *n* = number of specimens sampled

Species	Mean	Max	*n*
*C. carlosdelarosai*	1.8%	4.6%	92
*C. rafamoralesi*	1.1%	2.9%	89
*C. trilineatum*	3.0%	3.0%	2
C. *chinantecum* sp. nov.	-	-	1

**Table 2:** Interspecific genetic distance (mean/min)

Species	*C. chinantecum* sp. nov	*C. trilineatum*	*C. rafamoralesi*
*C. chinantecum* sp. nov	-		
*C. trilineatum*	6.19/5.09%	-	
*C. rafamoralesi*	11.68/11.32%	9.05/8.06%	-
*C. carlosdelarosai*	7.32/5.86%	6.58/5.28%	10.58/8.89%

**Table 3:** Comparison of fixation index (*FST*) between species pairs

Species 1	Species 2	*FST*
*C*. *chinantecum* sp. nov.	*C. trilineatum*	0.68
*C*. *chinantecum* sp. nov.	*C. rafamoralesi*	0.92
*C*. *chinantecum* sp. nov.	*C. carlosdelarosai*	0.82
*C. trilineatum*	*C. rafamoralesi*	0.69
*C. trilineatum*	*C. carlosdelarosai*	0.53
*C. rafamoralesi*	*C. carlosdelarosai*	0.81

**Fig. 3:**
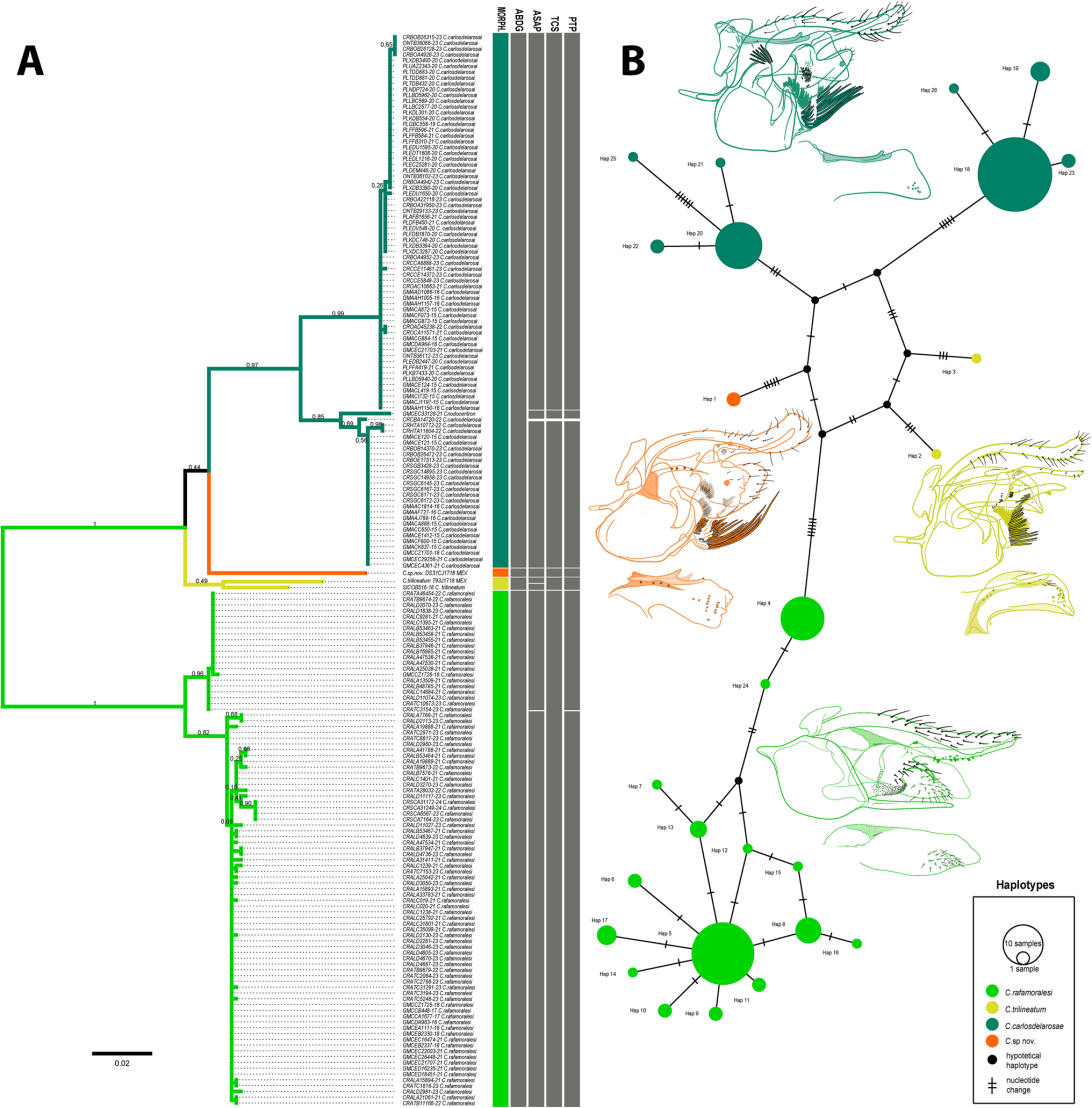
Phylogenetic placement and haplotype network: **A** Molecular analysis. Maximum likelihood tree obtained using mitochondrial cytochrome c oxidase subunit I (COI) sequences (649 bp) and species delimitation by morphology and molecular delimitation methods. Values represent ML bootstrap replications (1000 replicates). **B** TCS haplotype network and morphology of respective male genitalia

The maximum likelihood tree (Fig. 3A) placed the new species as sister to *C. carlosdelarosai* (45% bootstrap support). Results of molecular species delimitation methods are also displayed in Fig. 3; only the model ABGD agreeing with morphology and confirming four nominal species. The model ASAP suggests that there may be eight species; the models TCS and PTP suggest that there could be six and seven species, respectively. The ranges of intraspecific and interspecific variation observed are indicated in Tables 1 and 2, respectively. A total of 26 haplotypes were identified among the four analyzed species (Fig. 3B): 15 from *C. rafamorales*i, 8 in *C. carlosdelarosai*, while few sequences were available for the new species and *C. trilineatum* to cover their populational genetic diversity.

## Taxonomy

*Caenocentron chinantecum* Razo-González and Vilarino, sp. nov. https://zoobank.org/NomenclaturalActs/1123C957-4600-407B-8E13-9BB65E579E5A. 

Figures 4 and 5

**Fig. 4:**
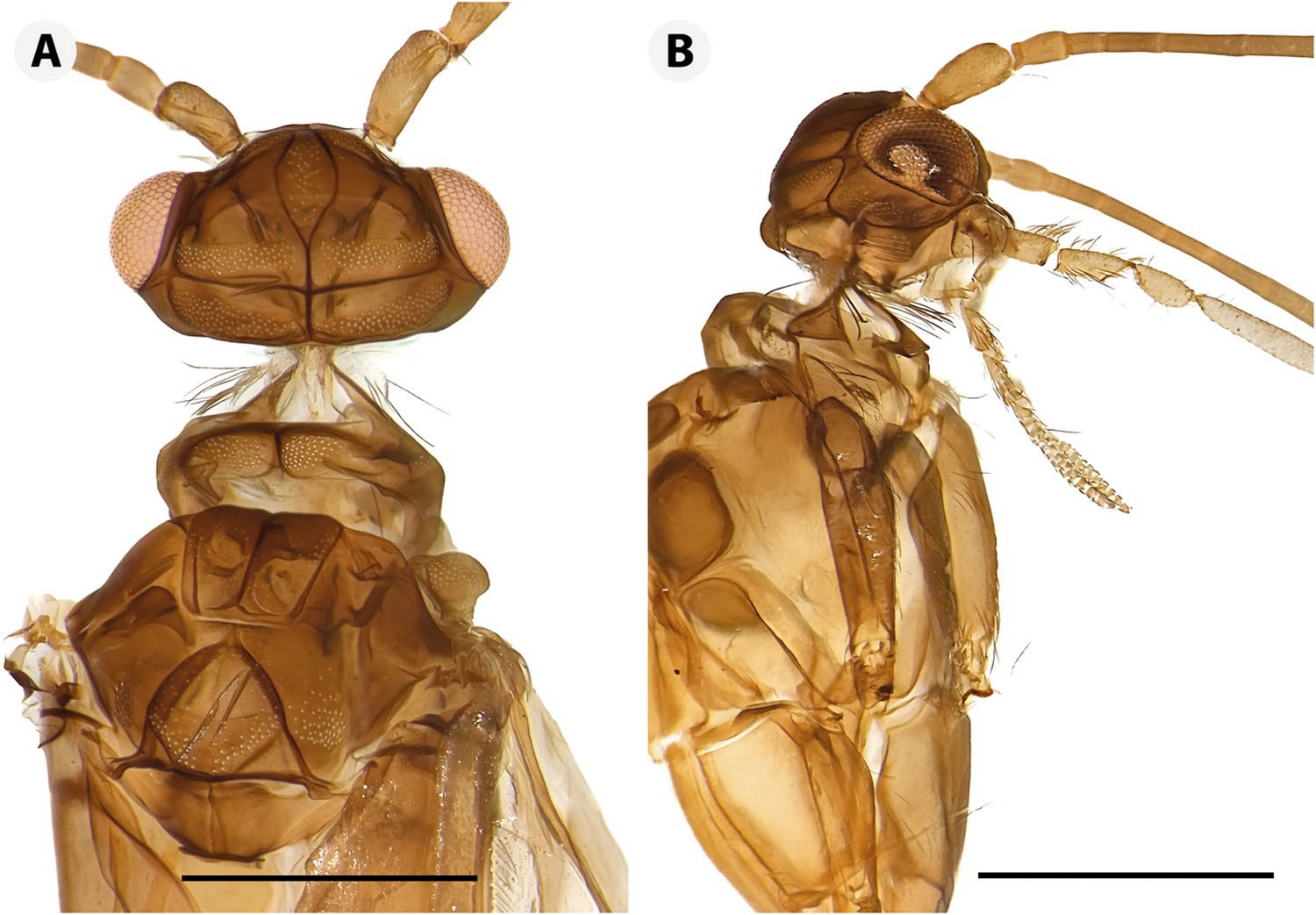
*Caenocentron chinantecum* Razo-González and Vilarino sp. nov., holotype male: **A** head and thorax dorsal view; **B** same lateral view showing palps. Scale bar = 0.5 mm

**Fig. 5:**
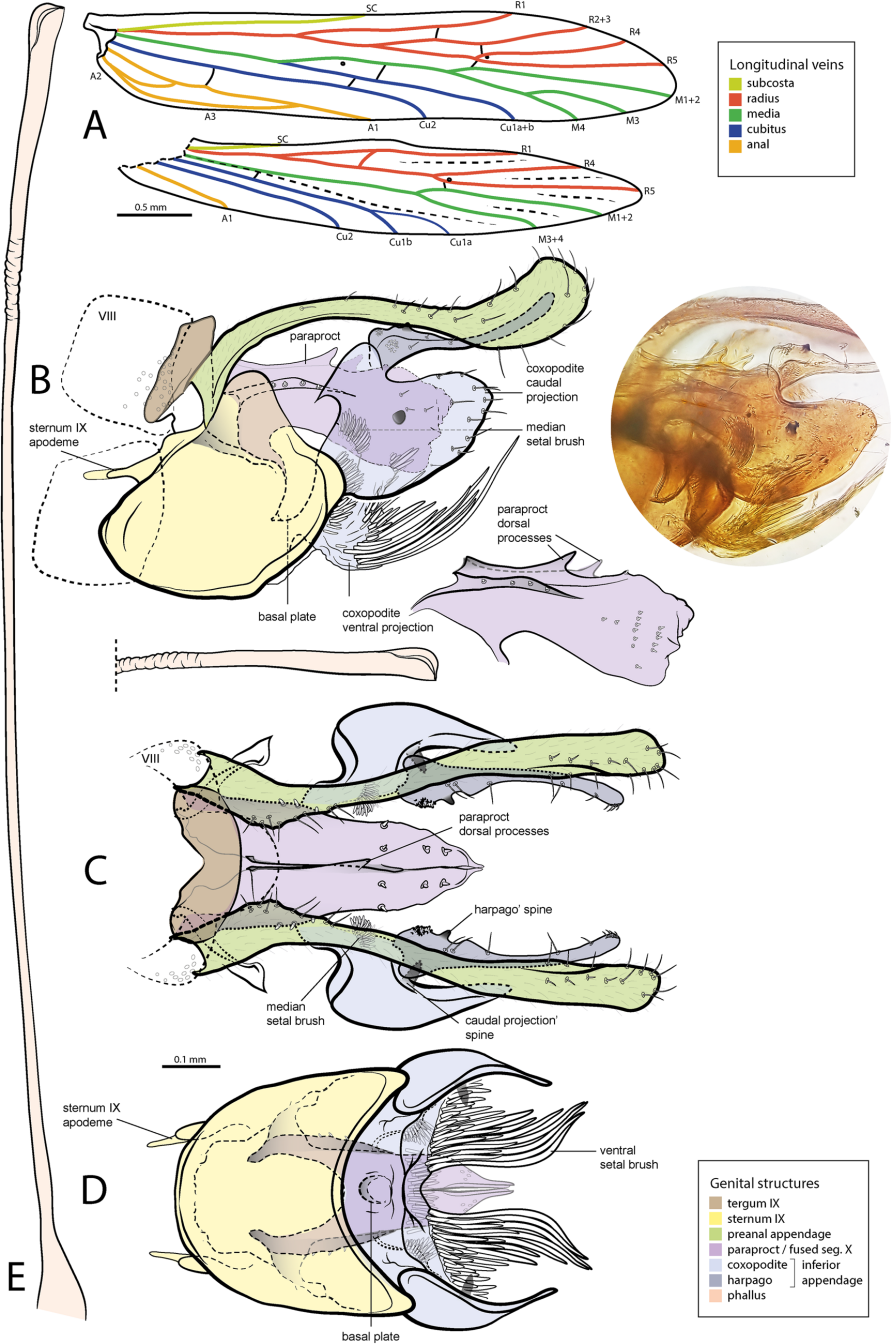
*Caenocentron chinantecum* Razo-González and Vilarino sp. nov. (holotype): (A) venation of the right forewing (above) and hindwing (below) (longitudinal veins highlighted in colors); (B) male genitalia, left lateral view, paraproct in detail, and photograph showing spines aspect lateral view; (C) male genitalia, dorsal view; (D) male genitalia, ventral view; (E) phallus in full length, left lateral view. Structures are indicated in different colors

**Diagnosis**. *Caenocentron chinantecum* sp. nov. is most similar to *C. carlosdelarosai*, mainly by the absence of stout spines on the ventral projection of coxopodite and may be distinguished from this species and other congeners by the following combination of characters: (1) spine of the caudal projection of coxopodite located nearly at coxopodite midlength; (2) caudal projection of coxopodite wider; (3) harpago basal region wide (more than 2 times as wide as apex); (4) presence of lateral strip with spine-like setae on paraproct; and (5) paraproct dorsal processes asymmetric; (6) preanal appendage base narrow.

**Description**. *Adult male* (Figs. 4 and 5): Forewing length 4.00 mm (*n* = 1). Color uniformly pale brown (in alcohol) (Fig. 4A, B). Maxillary palp formula (I = II = III) < IV < V; segment IV shorter than sum of segments I-II-III (Fig. 4B); tibial spurs 2–4−3, male hindleg apical spur unmodified. Forewing (Fig. 5A) fork II and IV present; fork II sessile; discoidal cell about as long as thyridial cell; three anal veins present. Hindwing (Fig. 5A) fork II and V present; fork II sessile; transverse vein between R1 and SR present. Abdominal sternum V with anterolateral oval region with cuticle modified and reticulate.

*Male genitalia* (Fig. 5). Tergum IX, in lateral view (Fig. 5B), narrow, height greater than length; in dorsal view (Fig. 5C), membranous mesally. Sternum IX, in lateral view (Fig. 5B), subquadrate, height greater than length; anterior apical margin truncate; dorsal margin prominent; in ventral view (Fig. 5D), apical margin concave. Segment X membranous, fused to paraproct. Paraproct, in lateral view (Fig. 5B), oblong, with sclerotized lateral strip bearing spine-like setae, dorsal margin sclerotized, with pair of asymmetric short processes, apex membranous; in dorsal view (Fig. 5C), mid-dorsal margin sclerotized, not fused, meeting at midlength. Preanal appendage, in lateral view (Fig. 5B), strongly sinuous, slender, base narrow, apex round, enlarged, about 3× as wide as midlength. Inferior appendage with coxopodite and harpago distinct. Coxopodite, in lateral view (Fig. 5B), median region with long setal brush and stout spine at midlength; caudal margin produced, caudal projection about 3 × as wide as midlength preanal appendage, apex subrounded; basal surface with long ventral projection, ventral projection with row of setae mesally and along its margin, apical setae longer; in ventral view (Fig. 5D), ventral projection with mesal setae longer and bent laterad. Harpago, in lateral view (Fig. 5B), wide at base, narrow at midlength and apex, basal third with short spine and group of several diminute setae; in dorsal view (Fig. 5C), basal third enlarged. Basal plate, in lateral view, directed ventrad; in ventral view (Fig. 5D), short. Phallus (Fig. 5E) tubular, long and slender, base flared, reaching segment V; apex slightly enlarged.

**Etymology**. The specific epithet refers to the culture and language of the indigenous people of the Chinantla region, where the holotype comes from.

**Material examined.**

**Holotype**. MEXICO • ♂ (CNIN:TC9108-DNA voucher); Oaxaca, Santiago Comaltepec, San Martín Soyolapam; San Martín Soyolapam river, 17°41′47″N 96°16′54″W, 136 m a.s.l.; 26.x.2016, R Novelo-Gutiérrez, JA Gómez-Anaya, M Razo-González.

**Distribution**. MEXICO: Oaxaca state.

**Remarks**.The morphology of *Caenocentron chinantecum* sp. nov. holds overall similarity with *C. carlosdelarosai* with a similar inferior appendage shape; however, the presence of lateral rows of spine-like setae on paraproct is a character only shared with *C. trilineatum*. The minimum genetic distance between *Caenocentron chinantecum* sp. nov. and *C. carlosdelarosai* is 5.86%, and between *C. trilineatum* 5.09%. The new species is indicated as a radiation between these two species.

***Caenocentron trilineatum* (**Mosely 1934**)**

*Melanotrichia trilineata* Mosely 1934: 140. [Type locality: Mexico, Teapa, Tabasco; BMNH; ♂]

*Xiphocentron trilineatum* – Bueno-Soria and Flint 1978: 197 [distribution to Guatemala and El Salvador].

*Cnodocentron* (*Caenocentron*) *trilineatum* – Schmid, 1982: 112 [checklist]. – Holzenthal and Calor 2017: 454 [catalogue].

*Caenocentron trilineatum* – Vilarino et al. 2022 [revision, phylogeny], – Razo-González et al. 2023 [distribution to Mexico, Oaxaca]

**Material examined**.

MEXICO • 1♂(CNIN-DNA voucher) Veracruz, San Andrés Tuxtla, arroyo Laguna Escondida, Los Tuxtlas Biological station, 17.xi.1993, R.W. Baumann • 4♂♂(CNIN) San Andrés Tuxtla, arroyo anexo La Palma, 21.vi.1977, F. Gonzales • 15♂♂(CNIN) San Andrés Tuxtla, Playa Escondida, E. Barrera, 27.iii.1976. • 5♂♂, 1♀(CNIN) Same except, 2.xii.1977, J. Bueno-Soria. • 1♂(CNIN) San Andrés Tuxtla, Salto de Eyipantla, 30.vii.1976, J. Bueno-Soria.

**Distribution**. El Salvador, Guatemala, Mexico (Oaxaca, Tabasco, Veracruz).

**Remarks**. The species distribution in Mexico is indicated to Veracruz state (Bueno-Soria and Flint 1978; Razo-González et al. 2023); however, no analyzed material is listed in any previous references. Herein we included the specimens analyzed from CNIN occurring in Veracruz.

### **Identification key to adult males of***Caenocentron***(modified from Desiderio et al. 2025)**

**1** Sternum IX with apical margin produced into elongate processes ……………..…………..…. **2**

**1**’ Sternum IX with apical margin not produced, concave ………………….…………….…….. **3**

**2** **(1)** Caudal projection of sternum IX forming two narrow, elongate processes ………………………………………………………………………………….…. ***C. rafamoralesi***

**2’** Caudal projection of sternum IX forming a broad, deltoid plate with two short apical processes ………………………………………………………………………………………….. ***C. yavapai***

**3 (1)** Coxopodite caudal margin projection narrow, acute …………………………………….......**4**

**3’** Coxopodite caudal margin projection broad ……………………………….……………...…... **5**

**4 (3)** Sternum IX in lateral view truncate apically; coxopodite in ventral view with ventral projection bearing long, dense setae along most of the inner margin, including mesal region; each coxopodite with one short subapical spine on inner face ………………………...… ***C. trilineatum***

**4’** Sternum IX in lateral view deltoid apically; coxopodite in ventral view with ventral projection bearing lateral setal brushes, mesal region glabrous; each coxopodite with two short spines on inner face …………………………………………...…….….……………………………….... ***C. ideolus***

**5 (3)** Coxopodite caudal margin with a short spine or multiple long spine-like setae ………….… **6**

**5’** Coxopodite caudal margin lacking spines ………………………………….…………….…… **10**

**6 (5)** Coxopodites broadly fused at base in ventral view; inner margin with brush of long setae; caudal margin bearing a short spine ……………………………….……………………………..... **7**

**6’** Coxopodites separate at base in ventral view; inner margin without setal brush; caudal margin with line of several long spine-like setae……..……………….......……………..…….... ***C. roosevelt***

**7 (6)** Coxopodite inner margin (ventral view) with a brush of long setae and a pair of long sublateral stout spines; the basal third of harpago is strongly enlarged …………………….... ***C. immaculatum***

**7’** Coxopodite inner margin with brush of long setae, lacking stout sublateral spines; basal third of harpago slightly or not enlarged ………….………………………………………………………… **8**

**8 (7)** Harpago base with a line of long setae; coxopodite caudal margin projection with a short spine located ventroapically…………………………………............…………………………… ***C. lausus***

**8’** Harpago base with a group of very short setae or without any setae; coxopodite caudal margin projection with a short spine located mesally or subapically ………………………….…………… **9**

**9 (8)** Harpago base narrow, without any setae; coxopodite caudal margin projection with a short subapical spine; paraproct in lateral view only one dorsal process is visible........ ***C. carlosdelarosai***

**9’** Harpago base wide, with a group of very short seta; coxopodite caudal margin projection with a short mesal spine; paraproct in lateral view two dorsal processes are visible..….……………….….……………………………………………………………………………….C. chinantecum sp. nov.

**10 (5) **Inferior appendage with lobe bearing brush of setae near harpago base; coxopodite median region with nearly indiscernible small setules; paraproct dorsal margin without process, ventroapical margin rounded …………..………………….……………………...……….. ***C. pallas***

**10’** Inferior appendage without brush of setae near harpago base; coxopodite median region with transverse patch of setae; paraproct dorsal margin with short, acute process, ventroapical margin acute …………..……………………………………………………………………..…… ***C. galesus***

## Discussion

Current knowledge about the diversity of Xiphocentronidae in the Neotropical region remains incomplete and is heavily biased toward areas that have historically been better sampled. Recent taxonomic studies in southeastern Oaxaca, Mexico, have revealed considerably greater diversity than previously recognized, including the description of new species and important extensions in their distribution range (Razo-González 2018; Razo-González et al. 2020, 2023). This highlights the importance of sampling efforts targeting poorly explored regions. Of the 11 species now recognized in the genus *Caenocentron*, only three are distributed in Mexico: *Caenocentron ideolus*, *C. trilineatum*, and *C. chinantecum* sp. nov. Unfortunately, *C. ideolus* is known solely from its type locality, which has since been absorbed by urban expansion, and the species has not been recorded in other regions, including nearby natural areas surrounding the type locality.

The degree of the species differentiation according to the FST (Table 3) indicates a high genetic differentiation between the species, ranging from 0.53 to 0.92, suggesting that a greater part of the total genetic variation is due to interspecific differences. The model ASAP overestimated the number of species analyzed, suggesting eight nominal species; the models TCS and PTP propose six and seven species. In this study, none of the models analyzed showed consistency in the number of species, suggesting cryptic genetic divergence within *C. carlosdelarosai*, *C. trilineatum*, and *C. rafamoralesi.* However, the morphology and the interspecific variation do not support the idea that they are different species, but species such as *C. carlosdelarosai* could be separated in two genetic lineages. The COI sequences of *C. carlosdelarosai* formed two clusters that correspond to two barcode index numbers (BINs) in BOLD (based on 3% K2P distance barcoding gap threshold). The maximum genetic distance between each cluster of *C. carlosdelarosai* was high (4.57%), which could explain why the models ASAP, TSC, and PTP suggest the existence of two species (Fig. 2). However, the specimens of these both genetic lineages are sympatric and at morphological level specimens from each cluster did not show any conspicuous difference in the male genitalia or other body structures. Therefore, they should be considered a single species. Maximum intraspecific genetic variation of other *Caenocentron* species was around 3%. The minimum interspecific differences were around 5%. The value of 5% may serve as a higher-confidence threshold for genetic species delimitation within the genus *Caenocentron*. Zhang and Bu (2022) showed that more than 30% of the Trichoptera have intraspecific genetic distances greater than 3%; therefore, the traditional threshold of 3% can overestimate the species diversity as occurred here with the species delimited by the ASAP, TSC and PTP.

The scope here was restricted to genetic comparison between the new species and a few other species with COI data available. According to the total evidence phylogeny (Vilarino et al. 2022), *C. carlosdelarosai* is closer to *C. lausus* (but with very low support). The new species has characters such as a paraproct with a sclerotized lateral band with spines shared by only *C. trilineatum*; they are genetically close (minimum interspecific distance of 5.09%) and their distribution overlaps. Therefore, it is likely that *C. chinantecum* sp. nov. would be a lineage between *C. trilineatum* and the southern species (*C. lausus* + *C. carlosdelarosai*). The inferred divergence between *C. lausus* and *C. carlosdelarosai* was in the Miocene ~7.9 mya, and between *C. trilineatum* and C. ideolus was ~7.8 mya; thus, *C. chinantecum*’s origin must also be around the Miocene.

From a biogeographic perspective, *C*. *lausus* and *C. carlosdelarosai* have a closer distribution, both in the Chortis block (Nicaragua, El Salvador, Honduras). This land block was connected to the Pacific coast of Mexico (states of Guerrero, Oaxaca) during the Paleocene until the early Eocene when it detached and moved south (Schaaf et al. 1995; Mann 2007; Silva-Romo et al. 2018), and collided with Yucatan along the Cayman trough in the Miocene (Coates et al. 2004). The biogeographic analysis indicated that the radiation of the genus occurred from this block (Vilarino et al. 2022). However, the distribution of the new species further north suggests that rather than being restricted to the Chortis block, the ancestral distribution of this lineage possibly extended from the Trans-Mexican Volcanic Belt to Costa Rica. This broader distributional hypothesis is consistent with the geological connectivity of Mexico and the Chortis block.

Our findings highlight the following: (1) Trichoptera species may have intraspecific genetic variation greater than 3%, and *Caenocentron* species delimitation threshold should be around 5% K2P distance; (2) the ancestral distributional range of this lineage possibly includes also Central Mexico and not only Central America; (3) Xiphocentronidae diversity is still undersampled and poorly studied regions may include new species that could change the understanding of the evolutionary history of the family; (4) this study provides new molecular information for *Caenocentron* species and increases the number of nominal valid species to 11.

## Data Availability

All data analysed during this study is available at public databases GenBank and BOLD.
